# Predicting glucocorticoid resistance in multiple sclerosis relapse via a whole blood transcriptomic analysis

**DOI:** 10.1111/cns.14484

**Published:** 2023-10-10

**Authors:** Maud Bagnoud, Jana Remlinger, Sandrine Joly, Marine Massy, Anke Salmen, Andrew Chan, Dimitris Karathanassis, Maria‐Eleptheria Evangelopoulos, Robert Hoepner

**Affiliations:** ^1^ Department of Neurology Inselspital, Bern University Hospital, University of Bern Bern Switzerland; ^2^ Department of Biomedical Research University of Bern Bern Switzerland; ^3^ Graduate School for Cellular and Biomedical Sciences University of Bern Bern Switzerland; ^4^ Department of Neurology, Eginition Hospital National and Kapodistrian University of Athens Athens Greece

**Keywords:** glucocorticoid, glucocorticoid receptor, multiple sclerosis, resistance

## Abstract

**Aims:**

Treatment of multiple sclerosis (MS) relapses consists of short‐term administration of high‐dose glucocorticoids (GCs). However, over 40% of patients show an insufficient response to GC treatment. We aimed to develop a predictive model for such GC resistance.

**Methods:**

We performed a receiver operating characteristic (ROC) curve analysis following the transcriptomic assay of whole blood samples from stable, relapsing GC‐sensitive and relapsing GC‐resistant patients with MS in two different European centers.

**Results:**

We identified 12 genes being regulated during a relapse and differentially expressed between GC‐sensitive and GC‐resistant patients with MS. Using these genes, we defined a statistical model to predict GC resistance with an area under the curve (AUC) of the ROC analysis of 0.913. Furthermore, we observed that relapsing GC‐resistant patients with MS have decreased *GR*, *DUSP1,* and *TSC22D3* mRNA levels compared with relapsing GC‐sensitive patients with MS. Finally, we showed that the transcriptome of relapsing GC‐resistant patients with MS resembles those of stable patients with MS.

**Conclusion:**

Predicting GC resistance would allow patients to benefit from prompt initiation of an alternative relapse treatment leading to increased treatment efficacy. Thus, we think our model could contribute to reducing disability development in people with MS.

## Graphical abstract

A whole blood transcriptomic analysis was performed to predict steroid resistance in multiple sclerosis relapse.

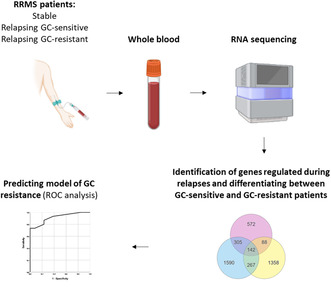

## 1 INTRODUCTION

Despite novel disease‐modifying treatment options, relapses still frequently occur in multiple sclerosis (MS). Indeed, the “Multiple Sclerosis in America 2017 survey” reported that more than 70% of participants experienced at least one relapse in the two years prior to the study start.^1^ More recently, data from the Swiss Multiple Sclerosis Registry showed that 13% of the overall relapsing–remitting MS (RRMS) patients experienced a relapse in the last six months.^2^ Relapses have been shown to increase the expanded disability status scale (EDSS) score by 0.5 points in more than 40% and by 1 point in almost 30% of the patients.^3^ Through impaired relapse recoveries within the first five years of disease, a faster transition from relapsing–remitting to a progressive phase can be noted, which increases not only the patient's disease burden^4^ but also leads to socioeconomic costs, that are even higher with more severe relapses.^5^, ^6^

Relapses are commonly treated with high‐dose intravenous glucocorticoid (GC)‐pulse therapy (i.e., 500–2000 mg methylprednisolone (MP) daily over 3–5 days).^7^ Although the exact mechanism of action remains imprecisely understood, GCs are known to induce both genomic and non‐genomic responses.^8^ They modulate the gene expression of different anti‐inflammatory cytokines^9^, ^10^ and alter signaling pathways involved in inflammation.^11^, ^12^ High doses of GCs enhance T cell apoptosis,^13^ reduce the expression of T cell adhesion molecules,^14^ and modulate the polarization and trafficking of monocytes.^15^ Clinically, intake of GCs shortens relapse duration, accelerates recovery, reduces the number of gadolinium‐enhancing lesions, and decreases disability.^16^ Side effects attributed to a short‐term high‐dose GC treatment have been reported in patients with MS, particularly in those with comorbidities such as diabetes mellitus, mood disorders, and cardiac abnormalities.^17^ These adverse effects are mostly mild and typically include headaches, insomnia, hot flashes, and anxiety.^18^, ^19^

Despite high‐dose GC therapy being the standard of care for acute MS relapses, 32% (intravenous GC) and 34% (oral GC) of patients in the North American Research Committee on Multiple Sclerosis (NARCOMS) register reported worse symptoms following steroid treatment than before their relapse.^20^ In another study, where GC resistance was defined as <1 point improvement in the affected functional systems scores (FSS) two weeks after GC administration, up to 75% of patients were considered GC refractory.^21^ Unfortunately, we cannot reliably predict if a patient will clinically respond to GC treatment. Predicting GC resistance in daily clinical care could significantly improve treatment efficacy and safety as it prompts physicians not to delay escalating treatment options. This study investigated the transcriptome of stable and relapsing MS patients with GC‐sensitive and GC‐resistant relapses.

## 2 MATERIALS AND METHODS

### 2.1 Cohort characteristics

The cohort included 15 stable RRMS patients (relapsing phenotype, no relapses or intravenous GCs within the last three months) and 28 RRMS patients during acute relapse (≤30 days since symptom onset; consisting of 13 relapsing GC‐sensitive and 15 relapsing GC‐resistant patients), all from the University Hospital Bern, Switzerland. From the Eginition University Hospital Athens, Greece, 22 relapsing RRMS patients (consisting of 21 relapsing GC‐sensitive and one relapsing GC‐resistant patients) were included. Relapse definition followed the current international standard criteria.^22^ Patients defined as GC‐responsive showed resolution of relapse symptoms and neurological signs within 4 weeks from the onset of relapse treatment. According to current guidelines in German‐speaking countries,^22^ patients with insufficient response to ≥2 high‐dose GC‐pulses (i.e., 500–2000 mg MP over 3–5 days) should undergo treatment intensification with plasma exchange within 4–6 weeks, if their EDSS has remained unchanged and the relapse acquired disability still affects activities of daily living. Taking all these considerations together, we defined clinical GC resistance as no change in EDSS after ≥1 high‐dose GC‐pulses (i.e., 500–2000 mg MP over 3–5 days) assessed 4 weeks after treatment initiation. Human studies were approved by the local authorities (Cantonal Ethic Committee Bern, Switzerland: 2017‐00060; Ethical Committee of Eginition University Hospital: 501/30.7.2019).

### 2.2 Sample collection

Blood from relapsing patients with RRMS and with GC‐sensitive and GC‐resistant relapses was collected before and/or after the first intravenous GC‐pulse administration, whereas blood from stable patients with RRMS was taken when stability was proven by no evidence of disease activity (NEDA)‐3 during the routine clinical visit. Blood was collected in 1 × 2.5 mL PAXgene blood RNA tubes (PreAnalytiX) to preserve the quality of the RNA. PAXgene blood RNA tubes were frozen and stored at −80°C. Blood was collected at both the University Hospital Bern and Eginition University Hospital Athens. The samples from the Eginition University Hospital Athens were sent to and processed at the University Hospital Bern.

### 2.3 RNA sequencing

RNA extraction and RNA sequencing were performed by the Next Generation Sequencing Platform of the University of Bern. Total RNA was extracted from whole blood stored in PAXgene blood RNA tubes using a Quick‐RNA Whole Blood kit (Zymo Research) according to the manufacturer's protocol. The recommended DNase treatment was included. The quantity and quality of the extracted RNA were assessed using a Thermo Fisher Scientific Qubit 4.0 fluorometer with the Qubit RNA BR Assay Kit (Thermo Fisher Scientific) and an Advanced Analytical Fragment Analyzer System using a Fragment Analyzer RNA Kit (Agilent), respectively. After that, the recovered RNA was used to make cDNA libraries using an Illumina Stranded Total RNA Prep with Ribo‐Zero Plus kit (Illumina) in combination with IDT for Illumina RNA UD Indexes set A (Illumina) strictly following Illumina's guidelines. The quantity and quality of the generated NGS libraries were evaluated using a Thermo Fisher Scientific Qubit 4.0 fluorometer with the Qubit RNA BR Assay Kit (Thermo Fisher Scientific) and an Advanced Analytical Fragment Analyzer System using a Fragment Analyzer RNA Kit (Agilent), respectively. Pooled cDNA libraries were paired‐end sequenced using NovaSeq 6000 S2 reagent Kit v1.5, 300 cycles (Illumina) on an Illumina NovaSeq 6000 instrument. The quality of the sequencing run was assessed using Illumina Sequencing Analysis Viewer (Illumina version 2.4.7). All the base call files were demultiplexed and converted into FASTQ files using Illumina bcl2fastq conversion software v2.20. Only samples of the University Hospital Bern cohort were sequenced.

### 2.4 RNA sequencing data analysis

Transcriptomic data were analyzed using Advaita Bio® software. Three hundred five genes regulated during relapse and differentiating between GC‐responsive and GC‐resistant MS relapses were identified. Afterward, receiver operating characteristic (ROC) curve analysis was run to stratify gene expression regarding their potential to separate GC‐resistant from GC‐responsive MS relapses, as described below. From these RNA sequencing data, differences in transcript isoform usage within the *GR* gene were also investigated.

### 2.5 Real‐time quantitative polymerase chain reaction (RT–qPCR)

The expression of *LDLRAP1*, *N4BP2L2*, *PGAP3*, *SNX2*, *ABHD8*, *AIM2*, *ANXA1*, *DDX54*, *EAF2*, *FASN*, *RHOT1*, *SNPH* genes was analyzed by RT–qPCR using the SYBR Green technology (LightCycler® 480 SYBR Green I Master, Roche). *ACTB* was used as a housekeeping gene. The sequences of the different primers are listed in Annex S1. Experiments were run on a light cycler 480 thermocycler and each reaction was done in triplicate. The relative gene expression was calculated as follows: Ct_target_/Ct_ACTB_. The University Hospital Bern cohort included leftover RNA from the RNA sequencing experiment and consisted of 10 relapsing GC‐sensitive and 15 relapsing GC‐resistant patients with MS. In addition, samples from 21 relapsing GC‐sensitive and one relapsing GC‐resistant patients with MS from the Eginition University Hospital Athens were added to the cohort. RNA extraction was done as described in previous sections. The quantity and quality of the extracted RNA were assessed using a NanoDrop microvolume spectrophotometer (Witec AG). cDNA was prepared from 100 ng RNA mixed with Quanta qScript cDNA SuperMix (VWR International). Reverse transcription was done using a thermal program of 5 min 25°C, 30 min 42°C, and 5 min 85°C. RNA was stored at −80°C.

### 2.6 Statistics

The statistical analysis of the transcriptomic experiment was performed by the Interfaculty Bioinformatics Unit (IBU) of the University of Bern. To consider the number of tests performed, a false discovery rate correction based on the procedure of Benjamini‐Hochberg was made. Using Advaita Bio® software, the transcriptomic data were analyzed to identify 305 genes regulated during relapse and differentiate between GC‐sensitive and GC‐resistant MS relapses. Afterwards, ROC curve analysis was run on IBM® SPSS® (version: 28.0.1.1) to stratify gene expression regarding their potential to separate GC‐resistant from GC‐sensitive MS relapses. Genes (*n* = 14) having an area under the curve (AUC) > 0.86 (*n* = 6) or <0.14 (*n* = 8) were further used for model generation. Of these 14 genes, two were non‐protein coding and therefore omitted. In the next step, transcriptomic results were confirmed by RT–qPCR. A ROC curve was run for each gene, and cut‐offs with at least a specificity of 80% (Table 1) were used. Genes upregulated in GC‐resistant relapses were scored with 1 if higher or equal to the cut‐off. Those being downregulated in GC‐resistant patients with MS were scored with 1 if lower than the ROC‐defined cut‐off (score 0–12 points). Afterwards, a second cohort of 21 relapsing GC‐sensitive and a single relapsing GC‐resistant patient sampled at a different University hospital (Eginition University Hospital Athens) were added to the initial cohort. The distribution of the score, as well as the cut‐off for the prediction of steroid resistance with at least a specificity of 80%, were set. Finally, this cohort was used for generating a logistic regression model being adjusted for age, sex, disease duration, immunotherapy, EDSS prior GC and dose of GCs used for relapse treatment; and within a second logistic regression model for the center.

**TABLE 1:** ROC‐defined cut‐offs of the RT–qPCR experiment using samples of the transcriptomic cohort (*n* = 10 relapsing GC‐sensitive and 15 relapsing GC‐resistant patients with MS).

Gene name	Cut‐off	Sensitivity	1—Specificity	AUC
*PGAP3*	1.587158	0.067	0.034	0.249
*LDLRAP1*	1.479333	0.067	0.033	0.316
*SNPH*	0.966720	0.244	0.200	0.479
*DDX54*	1.806113	0.067	0.067	0.270
*ABHD8*	1.489475	0.111	0.200	0.353
*FASN*	1.555318	0.067	0.138	0.253
*SNX2*	1.216048	0.489	0.200	0.638
*RHOT1*	1.507539	0.467	0.200	0.704
*EAF2*	1.297566	0.489	0.200	0.631
*ANXA1*	1.123340	0.556	0.200	0.730
*AIM2*	1.306647	0.844	0.200	0.808
*N4BP2L2*	1.295575	0.200	0.200	0.627

Abbreviation: AUC, area under the curve.

## 3 RESULTS

### 3.1 Cohort description

Characteristics of the pooled cohorts are displayed in Table 2. EDSS at baseline was not different between relapsing GC‐resistant and relapsing GC‐sensitive patients (*p* = 0.83; Table 2). However, EDSS after GC treatment was higher in relapsing GC‐resistant than in relapsing GC‐sensitive patients (*p* < 0.001; Table 2). Detailed baseline characteristics of the Bern and Athens cohorts are shown in Annex S2.

**TABLE 2:** Baseline characteristics of stable, relapsing GC‐sensitive, and relapsing GC‐resistant patients of the whole cohort, including the initial transcriptomic and the Eginition University Hospital Athens cohorts.

Variable	Stable (*n* = 15)	GC‐sensitive (*n* = 34)	GC‐resistant (*n* = 16)	*p*‐Value (sens vs. res)
Age (mean, min–max)	33.9 (17–51)	35.3 (18–64)	34.8 (23–57)	1.0
Sex (female)	11/15	25/34	12/16	0.91
Disease duration (mean, min–max)	4.6 (0–12)	4.1 (0–19)	1.6 (0–13)	0.05
EDSS prior GC (median, min–max)	1.5 (0–3.5)	2.5 (1.0–6.0)	2.89 (1.5–5.0)	0.83
EDSS post GC (median, min–max)	Not applicable	1.5 (0.0–5.0)	3.25 (1.5–5.0)	<0.001
GC dose used (mg)	0 (0–0)	4956 (1500–13,000)	5794 (1200–11,000)	0.33
*Immunotherapy*				
None	0	18	12	0.15
Mild‐to‐moderate efficacy	7	10	4	
High efficacy	8	6	0	

*Note*: Statistic: Chi2 and Mann Whitney *U* test. Classification of immunotherapy: none: no treatment prior relapse, mild to moderate efficacy: interferon beta formulations, glatiramer acetate, dimethyl fumarate, teriflunomide; high efficacy: anti‐CD20 (ocrelizumab, rituximab, ofatumumab), natalizumab and fingolimod.

Abbreviations: EDSS, expanded disability status scale; GC, glucocorticoid; max, maximum; min, minimum.

### 3.2 Stable, relapsing GC‐sensitive, and relapsing GC‐resistant MS patients do not show any differences in the expression levels of the GR isoforms

**FIGURE 1:**
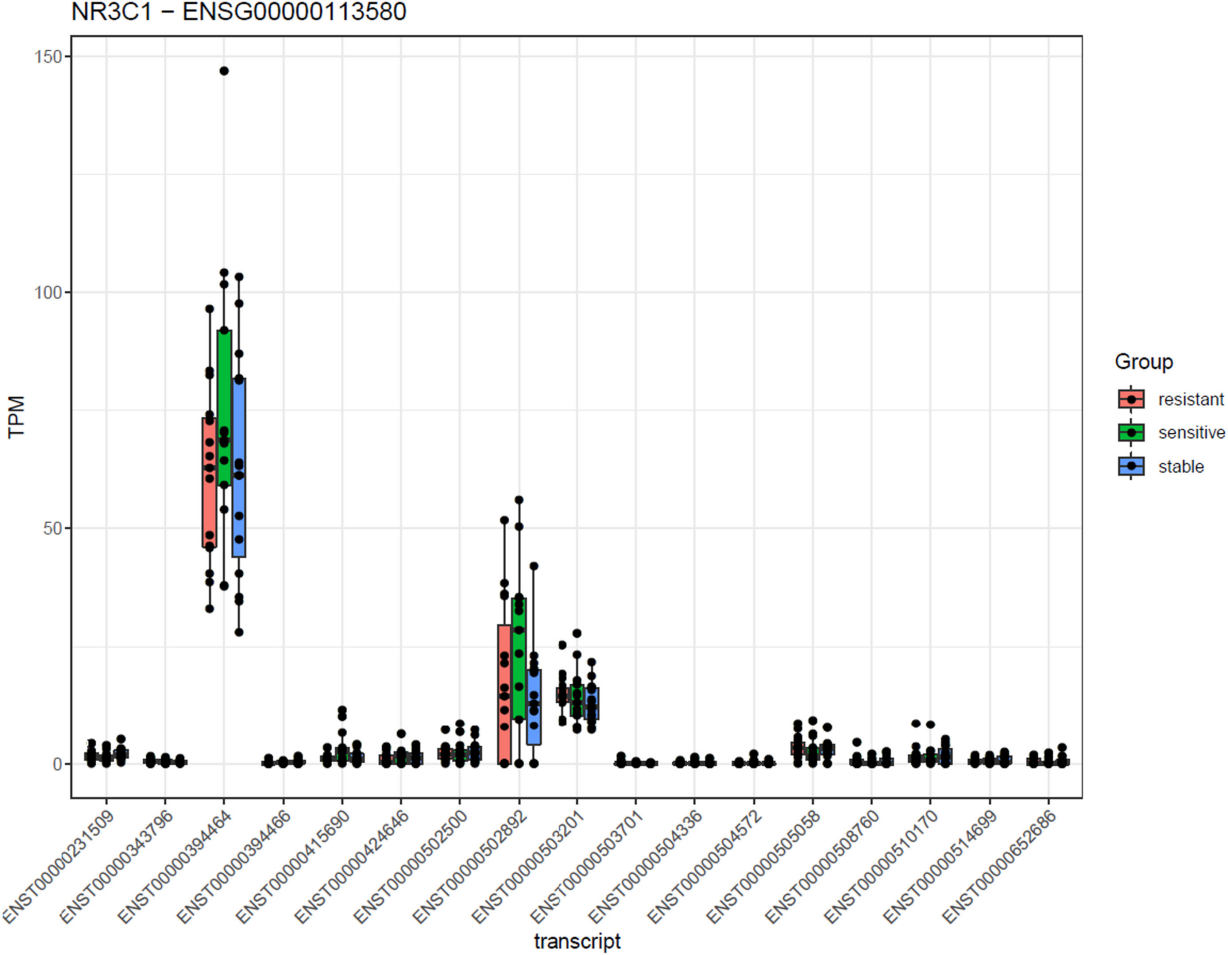
Blood transcriptome was analyzed in stable (*n* = 15), relapsing GC‐sensitive (*n* = 13), and relapsing GC‐resistant (*n* = 15) patients with MS, all coming from the University Hospital Bern, Switzerland. Differences in transcript isoform usage (DTU) within the *GR* (*NR3C1*) gene were investigated. The transcript IDs of the GR isoforms are represented on the *x*‐axis, whereas the *y*‐axis represents the transcript expression levels. RNA sequencing. Statistic: Two‐factorial model testing for differences between groups, accounting for sex (test performed with DEXSeq v. 1.36.0); not significant. NR3C1: glucocorticoid receptor; TPM: transcripts per million.

Differences in transcript isoform usage within the *GR* gene, the so‐called *NR3C1* gene, were investigated. The analysis demonstrated that none of the different isoforms investigated, including GRα and GRβ, significantly differ in their expression levels between the different MS groups (Figure 1).

### 3.3 Transcriptomic differences between relapsing GC‐sensitive and relapsing GC‐resistant patients with MS

**FIGURE 2:**
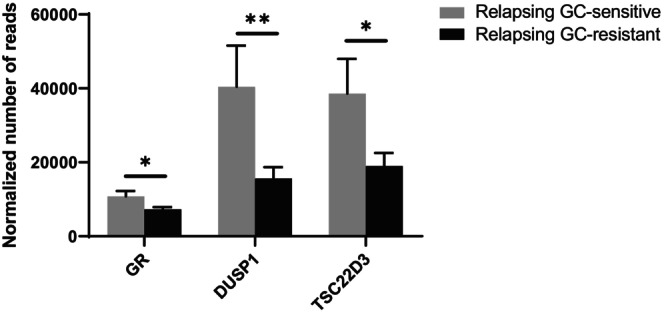
Blood transcriptome was analyzed in relapsing GC‐sensitive (*n* = 13) and relapsing GC‐resistant (*n* = 15) patients with MS, all coming from the University Hospital Bern, Switzerland. The expression of the *GR* and the two GR‐induced genes, *DUSP1* and *TSC22D3*, was analyzed in the two different groups of patients. RNA sequencing. Statistic: Correction for multiple testing according to the procedure of Benjamini–Hochberg; * *p* < 0.05, ** *p* < 0.01.

Our transcriptomic analysis reveals significantly reduced blood expression levels of the *GR* itself and the two GR‐induced genes, *DUSP1* and *TSC22D3* in relapsing GC‐resistant as compared to relapsing GC‐sensitive patients with MS (Figure 2).

### 3.4 Differentially expressed genes between stable, relapsing GC‐sensitive and relapsing GC‐resistant patients with MS

**FIGURE 3:**
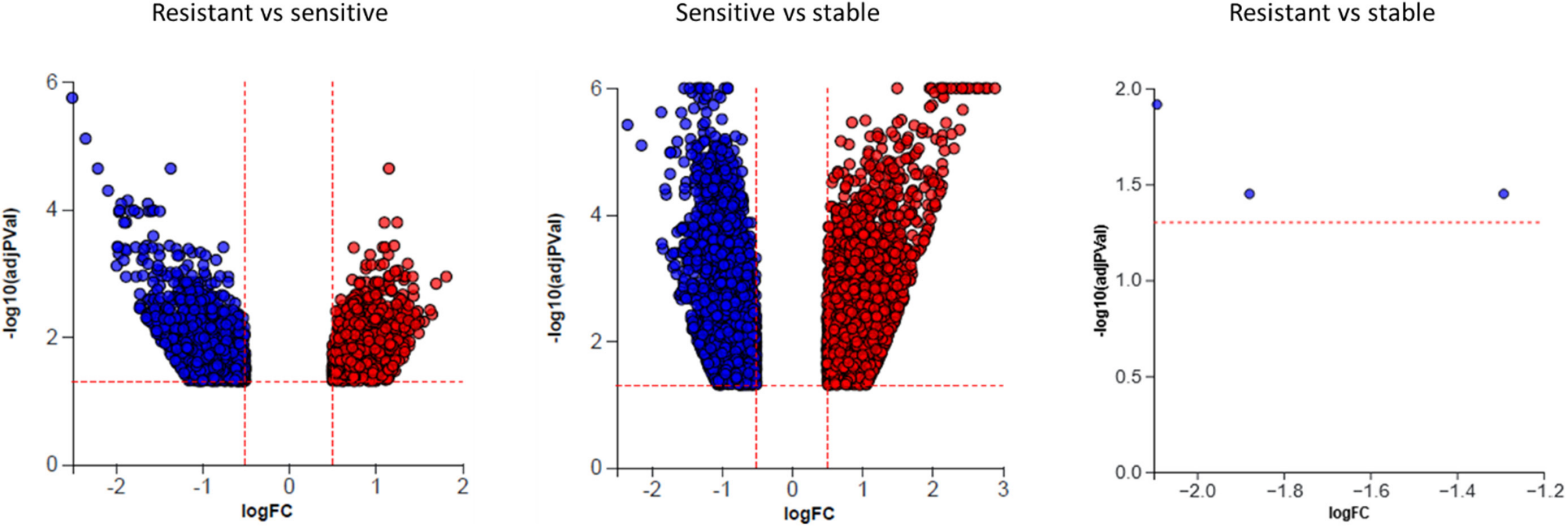
Blood transcriptome was analyzed in stable (*n* = 15), relapsing GC‐sensitive (*n* = 13), and relapsing GC‐resistant (*n* = 15) patients with MS, all coming from the University Hospital Bern, Switzerland. Differentially expressed genes are represented in terms of their measured expression change (*x*‐axis) and the significance of the change (*y*‐axis). The significance is represented in terms of the negative log (base 10) of the *p*‐value. The dotted lines represent the thresholds used to select the differentially expressed genes: 0.5 for expression change and 0.05 for significance. The up‐regulated genes (positive log fold change) are shown in red, while the down‐regulated genes are in blue. RNA sequencing. Statistic: Correction for multiple testing according to the procedure of Benjamini–Hochberg; adjusted *p*‐value <0.05. Graphics have been done by Advaita Bio® software.

The overall amount of differentially expressed genes among stable, relapsing GC‐sensitive, and relapsing GC‐resistant patients with MS is visualized through three volcano plots (Figure 3). In total, 2304 genes were differentially expressed between relapsing GC‐sensitive and relapsing GC‐resistant patients with MS, 4625 between relapsing GC‐sensitive and stable patients with MS, and three between relapsing GC‐resistant and stable patients with MS.

### 3.5 A predictive model of GC resistance

The following genes were identified by transcriptomic analysis: *PGAP3*, *LDLRAP1*, *SNPH*, *DDX54*, *ABHD8*, *FASN*, *SNX2*, *RHOT1*, *EAF2*, *ANXA1*, *AIM2*, and *N4BP2L2*. Using the transcriptomic samples, cut‐offs were defined by ROC in the RT–qPCR experiment (Table 1). Pooling these samples with additional patients from an independent University (Eginition University Hospital Athens) resulted in the final cohort of 47 patients. Here, the cut‐off of ≥8.8 provided a specificity of 84% and a sensitivity of 35% for predicting clinical GC resistance. Further, a logistic regression analysis to predict GC resistance adjusted for relevant confounders identified the score as a significant predictor [OR (95% CI) 1.8 (1.1–2.9); Table 3]. This holds true even after additionally adjusting for recruitment center—knowing that most relapsing GC‐resistant patients were included at the University Hospital Bern, Switzerland (OR (95% CI, *p*‐value) 1.75 (1.02–3.00, 0.04)).

**TABLE 3:** Logistic regression analysis to predict GC resistance in relapsing MS patients.

Variable	Odds ratio	95% CI LL	95% CI UL	*p*‐Value
Score	1.79	1.10	2.91	0.02
Female sex	1.44	0.23	9.10	0.70
Age (years)	0.98	0.90	1.06	0.53
Disease duration (years)	0.82	0.60	1.13	0.22
Immunotherapy group	0.34	0.08	1.51	0.16
EDSS prior GC	1.73	0.74	4.05	0.21
GC dose (mg)	1.00	1.00	1.00	0.09

Abbreviations: 95% CI, 95% confidence interval; EDSS, expanded disability status scale; GC, glucocorticoid; LL, lower limit; mg, milligram; UL, upper limit.

## 4 CONCLUSIONS

In this study, we analyzed the whole blood transcriptome of stable, relapsing GC‐sensitive, and relapsing GC‐resistant patients with MS in order to build a predictive model of GC resistance. We thus identified 12 genes regulated during a relapse and differentially expressed between relapsing GC‐resistant and relapsing GC‐sensitive patients with MS. These genes defined a statistical model to predict GC resistance with an AUC of the ROC analysis of 0.913. This model was confirmed including relevant confounders within a multivariable logistic regression model using the initial cohort extended by an additional cohort.

Others already used transcriptomic data, together with machine learning, to identify the disease stage of MS patients.^23^ In our group, we developed a model based on clinical data at the time of relapse to predict the patient‐specific amount of GCs that warrants a sufficient therapeutic response.^24^ In the same study, we defined, with a specificity of 60.5% and a sensitivity of 75.5%, a threshold dose at which treatment intensification would be needed and observed that serum calcidiol level and optic neuritis were independent predictors of the required GC dose.^24^ In vitro, assays such as the BrdU incorporation in lymphocyte steroid sensitivity (BLISS) assay, used to assess cell proliferation, and the dexamethasone suppression of lipopolysaccharide‐stimulated cytokine production (DSCP) test have been proposed to measure GC sensitivity as well.^25^, ^26^ In this study, we used gene expression analysis to predict GC resistance. The generated model is based on the expression of 12 genes and shows a specificity of 84% and a sensitivity of 35%. The clinical implication of such a model could improve the outcome and reduce the burden of relapsing patients with MS. Because it allows prompt initiation of the treatment escalation with plasma exchange regimens and thereby avoiding side effects due to unnecessarily prolonged and high‐dose GC treatments, not only efficacy but also safety of MS relapse treatment could be improved. Furthermore, independence of clinical, disease‐specific parameters makes our model potentially applicable for other chronic diseases, such as asthma, inflammatory bowel disease, lupus erythematosus, rheumatoid arthritis, and cancer in which GC resistance causes treatment challenges as well.^27^

Mechanistically, several gene and molecular alterations have been proposed to explain GC resistance. In the present study, we investigated differences in transcript isoform usage within the *GR* gene in the blood of stable, relapsing GC‐sensitive, and relapsing GC‐resistant patients with MS. More particularly, we looked at the two main GR isoforms, namely GRα—which is known as the classical active GR‐ and GRβ—which functions as a dominant negative inhibitor of the GR pathway. In addition, we analyzed other isoforms, such as GRγ. Increased GRβ levels have already been associated with GC resistance in asthma and inflammatory bowel disease.^28^, ^29^ A positive correlation between experimental autoimmune encephalomyelitis (EAE, the animal model of MS) disease severity and GC resistance and a down‐regulation of *GRα* mRNA expression in T cells of EAE diseased mice have been reported.^30^ Our analysis demonstrated no significant differences in transcript isoform usage within the *GR* gene between the different MS populations. However, we observed a reduced *GR* gene expression level in the blood of relapsing GC‐resistant patients with MS compared to relapsing GC‐sensitive patients with MS. This confirms previous data of our lab and others, demonstrating a decreased expression of GR protein in CD8^+^ T cells of relapsing GC‐resistant as compared to relapsing GC‐sensitive patients with MS^31^ and a reduced level of GR protein in total peripheral blood mononuclear cells (PBMCs) of relapsing GC‐resistant patients with MS.^32^ We also observed a reduced expression of the GR‐induced genes *DUSP1* and *TSC22D3* in the blood of relapsing GC‐resistant patients with MS compared to relapsing GC‐sensitive patients with MS, as demonstrated by others.^33^, ^34^ We want to mention other proposed mechanisms explaining GC resistance, for which further investigation was beyond the scope of this article but might warrant future studies: Polymorphisms and mutations in the *GR* gene, leading to a loss‐of‐function of the receptor, have been shown to impact GC sensitivity.^35^, ^36^ Signaling molecules and post‐translation modifications of the GR also contribute to GC resistance. Indeed, activation of the c‐Jun N‐terminal kinase (JNK) pathway leads to the inhibition of GR‐mediated transcriptional activation and increases GR nuclear export in a GR phosphorylating‐dependent manner.^37^, ^38^ Ubiquitination and the subsequent proteasome machinery are essential for GR protein turnover and potentially influence GC responsiveness.^39^ Interestingly, a T helper subset has also been linked to GC resistance. It was shown that Th17.1 cells, which have an increased multidrug resistance 1 (MDR1) and a reduced GR expression, exhibit a GC‐resistant phenotype.^40^ The sensitivity to GCs of these cells could be enhanced by vitamin D treatment.^41^

Transcriptomic analysis of different cell types, fluids, or tissues of MS patients has already been performed.^42^ In most studies, a dysregulation of the immune system has been observed.^42^, ^43^ With hundreds of genes being differentially expressed in T cells and monocytes from patients with MS compared to healthy controls,^44^ clinical implication of this data remains challenging. In the context of MS relapses, GR agonists were identified as the top drug class to restore gene transcription in these cells, suggesting an impairment of the GC response in relapsing patients with MS. In contrast to our results, they did not observe any changes in *GR* gene expression but rather in the gene expression of proteins involved in forming the GR complex.^44^ As mentioned, GCs are known to modulate gene transcription strongly and GC treatment was found to alter the transcriptome of nine different human hematopoietic and non‐hematopoietic cell types.^45^ In their study, Franco et al. observed that approximately 17% of the transcriptome is differentially expressed after GC treatment. Moreover, they noticed that the number of genes and the specific genes responding to GC are cell‐type specific, as only 0.3% of the genes had an altered expression in all cell types.^45^ We demonstrated that the transcriptome of relapsing GC‐sensitive and relapsing GC‐resistant patients with MS strongly differs, as 2304 genes are differentially expressed between these two groups. Interestingly, 4625 genes were differentially expressed in relapsing GC‐sensitive patients with MS compared to stable patients with MS. In contrast, we found only three genes to be regulated differentially in relapsing GC‐resistant patients with MS compared to stable patients with MS. Considering that GC‐resistant patients appear to have the same transcriptomic pattern as stable patients with MS, despite clinically and radiologically proven neuroinflammation in the sampled relapse‐cases, our data potentially explains GC resistance and might bear therapeutic consequences.

This study has several limitations. This is a retrospective study and we do have a center bias, as most of the relapsing GC‐resistant patients with MS and all stable patients with MS were included at the University Hospital Bern. Only one patient from the Eginition University Hospital Athens cohort fulfilled clinical criteria of GC resistance, hindering us to use this cohort as a validation cohort. These patients were added to our Bern cohort to increase the variation and therefore the generalizability of the analysis. The main logistic regression model remains significant after adjustment for this confounder. Differences in high effective treatment category and disease duration might influence the analysis as well. However, they were both included in the logistic regression model to control for it. Nevertheless, we recognize that the GC‐resistant group contains more patients with no MS immunotherapies, which should be considered when interpreting our data even though formally not significant. Further studies, including more patients, are needed to validate our predictive model of GC resistance.

## FUNDING INFORMATION

This work was supported by the Swiss National Foundation (no. 310030_172952), the Research Grant Diversity and Excellence in Basic and Translational Research from the Inselspital, Bern University Hospital and the Burgergemeinde Bern.

## CONFLICT OF INTEREST STATEMENT

SJ, MM, and DK do not declare any conflicts of interest. MB and JR are current employees of CSL Behring, not related to this study. AS: received speaker honoraria for activities with Bristol Myers Squibb, CSL Behring, Novartis, and Roche, and research support by the Baasch Medicus Foundation, the Medical Faculty of the University of Bern and the Swiss MS Society, all not related to this work. AA: has received speakers'/board honoraria from Actelion (Janssen/J&J), Almirall, Bayer, Bio‐gen, Celgene (BMS), Genzyme, Merck KGaA (Darmstadt, Germany), Novartis, Roche, and Teva, all for hospital research funds; he received research support from Biogen, Genzyme, and UCB, the European Union, and the Swiss National Foundation; he serves as associate editor of the European Journal of Neurology, on the editorial board for Clinical and Translational Neuroscience, and as topic editor for the Journal of International Medical Research. M.E. Evangelopoulos has received travel grants and consulting fees from Biogen, Teva, Merck, Roche, and Sanofi. All the conflicts are not related to this work. RH received speaker/advisor honorary from Merck, Novartis, Roche, Biogen, Alexion, Sanofi, Janssen, Bristol‐Myers Squibb, Teva/Mepha, and Almirall. He received research support within the last 5 years from Roche, Merck, Sanofi, Biogen, Chiesi, and Bristol‐Myers Squibb. He also received research grants from the Swiss MS Society and is a member of the Advisory Board of the Swiss and International MS Society. He also serves as deputy editor‐in‐chief for the Journal of Central Nervous System Disease. All the conflicts are not related to this work.

## CONSENT

All persons gave their informed consent prior to their inclusion in the study.

## Supporting information


Appendix S1.

## Data Availability

Data are available via corresponding author on reasonable request.
