# Supplementary material for: Predicting glucocorticoid resistance in multiple sclerosis relapse via a whole blood transcriptomic analysis
Source: CNS Neurosci Ther. 2023 Oct 10;30(2):e14484. doi: 10.1111/cns.14484 (PMC10848073; doi:10.1111/cns.14484)
Supplement: Supplementary file 1 — Appendix S1. [file CNS-30-e14484-s001.docx]

**Supplementary materials**

**Annex 1:** List of the primer sequences used for the study.

| **Gene** | **Full gene name** | **Forward sequence** | **Reverse sequence** |
| --- | --- | --- | --- |
| *LDLRAP1* | Low Density Lipoprotein Receptor Adaptor Protein 1 | CCATCAAGAGGATCGTGGCTAC | GGACACGTTCTCAATGAGCTGG |
| *N4BP2L2* | NEDD4 Binding Protein 2 Like 2 | CAGACAGGTTTGTGAACCAGCAG | GCCATCACGATTCTGACCAAGC |
| *PGAP3* | Post-GPI Attachment To Proteins Phospholipase 3 | CGCCAGCCAATCTACATGAGTC | CTGAGGCACTTTGTGACCTTCC |
| *SNX2* | Sorting Nexin 2 | TGAGGATGGTGAACAAGGCTGC | CAGACCAAGGCTTCAACACTGAC |
| *ABHD8* | Abhydrolase Domain Containing 8 | AGCCTACACCTTCTATGCGCTG | GCACTAGGTCTGGGTACTCATG |
| *AIM2* | Absent In Melanoma 2 | GCTGCACCAAAAGTCTCTCCTC | CTGCTTGCCTTCTTGGGTCTCA |
| *ANXA1* | Annexin A1 | GCGAAACAATGCACAGCGTCAAC | CAACCTCCTCAAGGTGACCTGT |
| *DDX54* | DEAD-Box Helicase 54 | CTGCTGGACAATGTCATCAACTAC | CAAGGAGTAGGCTGTGCCACTT |
| *EAF2* | ELL Associated Factor 2 | CCTTCCACACTGTGCGCTATGA | GGCAGAGTTATGGTCACCTGTTC |
| *FASN* | Fatty Acid Synthase | TTCTACGGCTCCACGCTCTTCC | GAAGAGTCTTCGTCAGCCAGGA |
| *RHOT1* | Ras Homolog Family Member T1 | GACAAAGACAGCAGGCTGCCTT | TCGCTGAACACTCCACACAGGT |
| *SNPH* | Syntaphilin | TCCTACAAGGGCAGTGACAGCA | GGATGCACACCTCCTTCTGCTG |
| *ACTB* | Actin Beta | CACCATTGGCAATGAGCGGTTC | AGGTCTTTGCGGATGTCCACGT |

Annex 1: List of the primer sequences used for the real time quantitative polymerase chain reaction (RT-qPCR) experiment.

**Annex 2:** Bern and Athens cohort description.

|  | GC-sensitive | | | GC-resistant | | |
| --- | --- | --- | --- | --- | --- | --- |
|  | Bern (n=13) | Athens (n=21) | P-value | Bern (n=15) | Athens (n=1) | P-value |
| Variable |  |  |  |  |  |  |
| Age (mean, min-max) | 31.3 (21-43) | 37.8 (18-64) | 0.18 | 35.5 (23-57) | 25 | n.a. |
| Sex (female) | 9 | 16 | 0.75 | 12 | 0 | n.a. |
| Disease Duration (mean, min-max) | 3.6 (0-19) | 4.4 (0-16) | 0.55 | 1.7 (0-13) | 0 | n.a. |
| EDSS prior GC  (median, min-max) | 2.5 (1-4) | 3 (1.5-6) | 0.22 | 3.0 (1.5-5) | 2.0 | n.a. |
| EDSS post GC  (median, min-max) | 2.0 (0-3) | 1 (0-5.5) | 0.14 | 3.0 (1.5-5) | 2.0 | n.a. |
| GC dose used (mg) | 4731  (1500-13000) | 5095  (5000-7000) | 0.12 | 5847  (1200-11000) | 5000 | n.a. |
| Immunotherapy |  |  | 0.28 |  |  | n.a. |
| None | 9 | 9 |  | 11 | 1 |  |
| Mild to moderate efficacy | 2 | 8 |  | 4 | 0 |  |
| High efficacy | 2 | 4 |  | 0 | 0 |  |

Annex 2: Baseline characteristics of relapsing GC-sensitive, and relapsing GC-resistant patients of the whole cohort, including the initial transcriptomic and the Eginition University Hospital Athens cohorts. Statistic: Chi2 and Mann Whitney U Test. Abbreviations: EDSS: expanded disability status scale, GC: glucocorticoid, max: maximum, min: minimum. Classification of immunotherapy: none: no treatment prior relapse, mild to moderate efficacy: interferon beta formulations, glatiramer acetate, dimethyl fumarate, teriflunomide; high efficacy: anti-CD20 (ocrelizumab, rituximab, ofatumumab), natalizumab and fingolimod..
